# Changes of intestinal microbiota composition and diversity in very low birth weight infants related to strategies of NEC prophylaxis: protocol for an observational multicentre pilot study

**DOI:** 10.1186/s40814-017-0195-y

**Published:** 2017-11-07

**Authors:** Stefan Kurath-Koller, Christine Moissl-Eichinger, Gregor Gorkiewicz, Raimund Kraschl, Claudia Kanduth, Barbara Hopfer, Berndt Urlesberger, Bernhard Resch

**Affiliations:** 1 Department of Pediatrics, Division of Neonatology, Medical University of Graz, Auenbruggerplatz 34/2, 8036 Graz, Austria; 2 Section of Infectious Diseases and Tropical Medicine, Department of Internal Medicine, Medical University Graz, Graz, Austria; 3 Institute of Pathology, Medical University of Graz, Graz, Austria; 4 Department of Pediatrics, General Hospital Klagenfurt am Wörthersee, Klagenfurt am Wörthersee, Austria; 5 Department of Pediatrics, General Hospital of Leoben, Leoben, Austria

**Keywords:** Preterm neonate, Microbiome, Necrotising enterocolitis, Microbiomics

## Abstract

**Background:**

At the Division of Neonatology, Department of Paediatrics, Medical University Graz, a unique regimen of necrotizing enterocolitis (NEC) prophylaxis in preterm infants showing a < 1500 g birth weight (i.e. very low birth weight, VLBW) is used. The regimen includes oral antibiotic and antifungal therapy and probiotic preparations as well as a standardised feeding regimen. The incidence of NEC in preterm infants treated by this regimen has been shown to be lower, reflecting 0.7% when treatment was initiated on the first day of life, compared to international incidence rates (5.1%). However, the impact of the prophylaxis regimen on the intestinal microbiome is yet unknown.

**Methods:**

We here report the protocol of an observational multicentre STROBE compliant pilot study in VLBW preterm infants. Research will compare three groups as defined by different NEC prophylaxis regimens. Each centre will provide 20 infants. Stool samples will be collected every 2 days throughout the first 2 weeks of life. Samples will be stored at − 80 °C until 16S-rRNA sequencing. 16S-rRNA genes will be amplified using suitable primers (specific for bacteria, fungi and archaea) and prepared for MiSeq Sequencing. Analyses will be performed using public analysis-pipelines, such as Mothur and Qiime, thus allowing an analysis of high-throughput community sequencing data. Abundance and composition changes in intestinal microbiota will be compared between the groups throughout the first 2 weeks of life.

**Discussion:**

Different surroundings at the three participating study centres, including contacts to care takers and parents, as well as feeding or medication all might influence intestinal microbiota composition and abundance. In the planned sequel study, this should be kept in mind and a more standardised process ought to be established. However, the results obtained from the presented pilot study will display the burden of bias and help to establish a more strict protocol for the future.

**Trial registration:**

Trial has been registered with the German Registry for Clinical Trials (registry ID DRKS00009290).

**Electronic supplementary material:**

The online version of this article (10.1186/s40814-017-0195-y) contains supplementary material, which is available to authorized users.

## Background

The genesis of the microbiome starts by vertical transmission of maternal microbiota, either during birth or even before delivery. Recent studies suggest the presence of a microbiome within the placenta and foetal meconium [1, 2]. The delivery mode has been found to significantly influence the intestinal colonisation of infants [3–7]. Data suggest that the development of the intestinal microbiome may also be influenced by genetics [8]. Intestinal colonisation patterns established within the first week of life are thought to have an impact on the composition of the individual’s future intestinal microbiota [6, 9–11]. In term infants, the intestinal microbiome undergoes rapid maturation throughout the first year of life and is considered to be securely established by the age of three. However, in preterm infants maturation is delayed. The individual intestinal microbiome of adulthood is likely governed by an interplay between initial colonising microbiota, genetics, gut development, diet, and environment [5, 12–14]. Nutrition, i.e. breastfeeding versus formula milk, plays a major role and alters early colonisation patterns of the neonatal intestinal microbiome. Breast milk contains several beneficial components, such as lactoferrin, or secretory IgA, this having antimicrobial and anti-inflammatory effects and stimulating growth of Bifidobacterium species (spp.) [15–17]. Human milk also contains live bacteria [18], and this, too, might contribute to the healthy gut microbiome development.

The intestinal microbiome interacts in a symbiotic relationship with its host. It contributes to nutrient utilisation by increasing digestive capacity and the ability to harvest nutrients from food. It supports vitamin biosynthesis and the production of other metabolites [12, 13, 19, 20]. In addition, the intestinal microbiome can limit nutrient resources available to pathogens, specifically by out-competing them for metabolic resources and physical space [20, 21]. Furthermore, development of barrier function, integrity, and immune function is aided by the intestinal microbiome.

### Intestinal microbiota in preterm infants

The intestinal microbiome of the preterm infant shows reduced microbial diversity with a concurrent increase in colonisation with pathogenic organisms [6, 8]. It is less stable compared to that of term born infants, and it shows delayed transition to an adult colonisation pattern [5, 12, 22, 23]. Gestational age may contribute to intestinal colonisation pattern [24]. Evolution of the preterm infants’ intestinal microbiome is marked by periods of abrupt population changes [25]. In preterm infants, facultative anaerobes such as *Enterococcus*, *Enterobacter*, and *Lactobacillus spp.*, as well as numbers of *Staphylococcus spp.* dominate while numbers of anaerobes like *Bifidobacterium*, *Bacteroides* and *Atopobium spp.* were found to be decreased [22–24]. Healthy breastfed term born infants were found to be colonised by *Bifidobacterium spp.* by day 7 of life while preterm infants were not [24]. Preterm infants exhibit an altered microbial diversity, and are both qualitatively and quantitatively immune compromised due to their underdeveloped immune system. An insufficient epithelial gut barrier function predisposes them to invasion by pathogens. These can trigger exaggerated inflammatory responses by the infants’ still developing immune system, which may lead to disease processes, such as NEC [7, 26]. Preterm infants’ immune dysfunction coupled with low diversity of intestinal microbiota and an overall predominance of pathogenic bacteria within the infants’ gut represents a prime example of dysbiosis [5, 27, 28].

### Necrotizing enterocolitis

NEC resembles the most common life-threatening emergency of the gastrointestinal tract in the neonatal period. The prevalence of NEC accounts for 7–11% in VLBW infants [29, 30], with a prevalence rate of 1–5% of infants in NICUs. NEC carries a mortality rate of up to 30%. Both incidence and case fatality rates increase with decreasing birth weight and gestational age, putting very low and extremely low (< 1000 g) birth weight infants at the highest risk. Although NEC is a multifactorial disease, primarily associated with intestinal immaturity. The concept of “risk factors” for NEC remains controversial. However, the greatest risk factor for NEC is prematurity [29, 30]. NEC probably results from an interaction between loss of mucosal integrity due to a variety of factors and the host’s response to that injury, leading to necrosis of the affected area. Various bacterial and viral agents have been recovered from cultures. However, in most situations, no distinct pathogen was able to be identified. NEC rarely occurs before enteral feeding is initiated and is much less common in infants that are fed human milk [31]. Aggressive enteral feeding may predispose infants to the development of NEC.

### Intestinal microbiota and NEC

Among risk factors, prematurity and accompanying intestinal colonisation represent the only consistently identified criteria [32]. Therefore, if prematurity is inevitable, colonisation of the infants gut resembles the major modifiable risk factor. Up to date, findings on intestinal colonisation and the microbiome aspect of NEC have shown to be inconsistent. While some studies have found a unique community structure of the intestinal microbiome in preterm infants prior to and at the time of disease onset [4, 5, 7, 21, 33], others have not [34, 35]. The intestinal microbiome of NEC-infants appears to first diverge from that of healthy controls as early as 3 weeks prior to disease [21]. Two weeks prior to onset of NEC, intestinal microbiota show increased amounts of *Proteobacteria spp.* while the *Bacterioidetes* spp. decrease [5]. Differences in intestinal microbiota abundance and diversity continue until onset of NEC, with infants having been characterised by an overall lower diversity index compared to the average preterm infant [33]. The time of onset of NEC (≤ 22 days of age or > 22 days of age) seems to discriminate abundance of intestinal microbiota [36]. Thus, it can be assumed that the specific infectious agent associated with NEC might vary by infants’ age at onset of disease.

### Aims and objectives

At the Division of Neonatology, Department of Paediatrics, Medical University of Graz, a unique regimen as prophylaxis of necrotizing enterocolitis (NEC) is used in preterm infants of < 1500 g birth weight [37]. The regimen includes oral antibiotic and antifungal therapy, probiotic drugs as well as a standardised feeding regimen and was established by the former head of neonatology despite a lack of evidence at the time. The incidence of NEC in preterm infants treated by this regimen has been shown to be lower, reflecting 0.7% when treatment was initiated on the first day of life [37], compared to international incidence rates (5.1%) [38]. By carrying out this study, we aim to provide estimates of differences in intestinal microbiota for future sample size estimation.

### Hypothesis

Differences in therapeutic and preventive approach to VLBW infants as defined by use of different preparations of probiotics, antibiotics, antifungal agents and feeding strategies might influence diversity and composition of gut microbiota. Thus, intestinal microbial diversity and abundance, as well as development of the intestinal microbiome within the first weeks of life in preterm infants should differ depending on the applied regimen. Differences regarding intestinal microbiota may be found that might additionally have long-term, up to now unknown, impacts on infant health and development. These differences may lead to the development of tailored probiotic formulas to improve NEC protection for the future.

Abundance and composition of intestinal microbiota might be found to differ between infants treated by the Graz NEC prophylaxis regimen and infants not receiving the prophylaxis regimen. Infants receiving the Graz NEC prophylaxis regimen might further show lower rates of *Proteobacteria* spp. and higher rates of germs used as probiotics and further aided by the consumption of human milk, such as *Bifidobacteria spp*.

The primary outcomes are intestinal microbiota composition and abundance, which will be summarised separately for each group in order to inform sample size estimation for a future definitive randomised trial. Secondary outcomes include hypothetical predominance or lack of specific species in infants having received the Graz NEC prophylaxis regimen.

## Methods and design

We propose a prospective controlled triple-centre cohort pilot study including preterm infants with a birth weight of < 1500 g treated at three NICUs with different regimens of NEC prophylaxis and feeding strategies. Data from the proposed project will be used to support planning and statistical power calculations for a larger sequel study.

To compare our infants with two other groups, we now co-operate with the Division of Neonatology, Department of Paediatrics, General Hospital of Klagenfurt, and the Division of Neonatology, Department of Paediatrics, General Hospital of Leoben, where different prophylactic approaches are performed. Regimens are shown in Table 1. Regimens used at the different centres are considered the standard care for each respective centre. At all three participating NICUs, a standardised feeding regimen is used, starting with breast milk as soon as possible and increasing feeding amounts throughout the first days of life. Fluid management (enteral and parenteral nutrition) depends on the birth weight (i.e. < 1000 g BW = 100 ml/kg/d, 1001-1499 g BW = 90 ml/kg/d, 1500–2499 g = 80 ml/kg/d, > 2500 g = 70 ml/kg/d). In infants > 1500 g birth weight, single feeds start at 5 ml at the first day of life, > 2500 g birth weight at 10 ml. If birth weight is below 1800 g and the infants’ mother is tested CMV IgG positive, pasteurised breast milk is used. If the mother lacks sufficient amounts of breast milk, pooled donor milk (accessible at NICU Graz only) and/or formula (accessible at all three centres) are fed. Fortifiers are used in infants < 1500 g birth weight, starting at the earliest when 50% of feeding amounts are tolerated orally. Number of included infants was calculated with help of statisticians. Twenty preterm infants per centre will allow reliable estimation of outcomes to inform a future definitive sample size calculation.

**Table 1:** NEC prophylaxis regimens

	NICU Graz	NICU Klagenfurt	NICU Leoben
Probiotics	Lactobacillus rhamnosus 1 g = 1 × 109 CFU/d p.o. split into 2 doses	Bifidobacterium infantis 2x109CFU/d and Lactobacillus acidophilus 2x109CFU/d in combination p.o.	None
Antibiotics	Gentamycin 7 mg/kg every 12 h p.o.	None	Gentamycin 7 mg/kg every 12 h p.o.
Antifungal agents	Nystatin 10.000 U/kg every 6 h p.o.	Fluconazol 6 mg/kg i.v. every 72 h (< 1000 g birth weight)	Nystatin 10.000 U/kg every 6 h p.o.
Feeding	Pooled or pasteurised BM; subsequent transition to mothers’ BM or preterm formula (hydrolyzed in case of birth weight < 1000 g)	Pasteurised BM (no pooled BM) or preterm formula	Pooled or pasteurised BM; subsequent transition to mothers’ BM or preterm formula (hydrolyzed in case of birth weight < 1000 g)

Legend: Table 1 displays NEC prophylaxis regimens used at the participating study centres at the Medical University Graz, General Hospital of Klagenfurt and General Hospital of Leoben

*BM* breast milk, p.o. per os, *CFU* colony forming unit, *U* units, *d* day, *g* gram, *kg* kilogramme, *mg* milligramme

### Study protocol

**Fig. 1:**
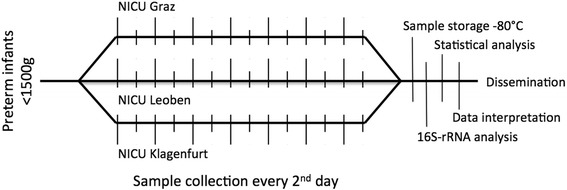
Study Protocol. Leg.: vertical lines illustrate sample collection dates; timeline is presented in days

Stool samples of 60 VLBW preterm infants, treated at the NICUs at the Department of Paediatrics, Medical University Graz (*n* = 20), the Department of Paediatrics, General Hospital of Klagenfurt (*n* = 20) and the Department of Paediatrics, General Hospital of Leoben (*n* = 20) will be collected every 2 days within the first weeks of life. Figure 1 shows the study protocol. Seven samples will be taken from each infant. Samples will be partitioned into two containers so that one container may remain stored for backup analysis. Prior to sample collection, feasibility of the DNA isolation method at the Core Facility for Molecular Biology, Medical University Graz was positively tested using six samples from infants of < 1500 g birth weight.

### Population

Very low birth weight preterm infants (i.e. < 1500 g birth weight).

*Inclusion criteria* comprise birth weight < 1500 g, treatment at the NICU, Department of Paediatrics, Medical University Graz; NICU, Department of Paediatrics, General Hospital of Leoben; NICU, Department of Paediatrics, General Hospital of Klagenfurt between October 2015, and March 2017.

*Exclusion criteria* comprise infants nonviable at birth, death within the first 2 weeks of life, infants suffering from genetic diseases, genetic syndromes or congenital anomalies, and meconium ileus.

Follow up: no follow up planned.

### Methodology

Infants will be recruited at all three centres to compare microbiota-analysis data between these groups. Prior to inclusion, infants’ parents are informed about the intention and purpose of the study, procedure of stool sampling, advantages, and disadvantages for their infant, and that the infant does not get harmed or treated differently for the purpose of the study. Parental information is provided by a written consent form, as well as by an attending paediatrician. Parents must give written informed consent before inclusion of the infant. Overall, seven stool samples, stored in two containers, of spontaneously produced faeces should be collected throughout 2 weeks, starting on the day of the infants’ first defecation, i.e. meconium loss. No additional interventions to stimulate defecation, deviating from standard care, are performed for study purposes. Collection of stool samples will be performed using sterile microspatula (SteriWare® Microspatula, Sampling Systems Ltd., UK) and sterile stool sample containers (CryoTube™ Vials, Thermo Fischer Scientific, Denmark). Up to 1 ml of faeces is taken. All samples taken will be allocated a running number. In order to record sample numbers and progress of sample collection, a standardised protocol-sheet is used. Storage of the samples taken will be performed using a deep-freezing storage device at − 80 °C as soon as possible. Within the freezer sample, containers are kept inside cardboard boxes (Fiberboard Cryo Box, Thermo Scientific™, Denmark) to protect them from any damage. Delivery of samples from Klagenfurt and Leoben will be performed by a professional delivery service using dry ice cooling and a temperature control protocol. After collection is completed, microbiome analysis will be performed.

We plan to include 20 infants of < 1500 g birth weight (VLBW) from each of the three participating study centres. This, in turn, means a number of 60 infants included in our study. Starting on the day of the infants’ first spontaneous stool, collection of seven samples, acquired every 2 days throughout the first weeks of life, is performed in every infant. Finally, all 420 samples will be analysed using 16S-rRNA-analysis techniques.

To provide high quality throughout all steps of the study protocol, all procedures as described above have been defined as standard operating procedures (SOP) for every centre.

The following data will be collected throughout hospitalisation, and noted in an excel sheet. For backup purposes, an additional hard copy containing the data will be deposited at each centre:

- *Maternal and pregnancy associated data*: mothers’ age, number of prior pregnancies, number of infants born, multiple pregnancy, existence of mother-child-passport (standardised protocol booklet to document pre-/peri- and postnatal findings; for detailed information please see https://www.help.gv.at/Portal.Node/hlpd/public/content/8/Seite.082201.html), pregnancy associated complications, application of steroids to induce lung maturation, tocolysis.
- *Perinatal data*: complications during birth, birth-mode, APGAR-score, umbilical cord pH.
- *Neonatal data*: sex, birth weight, small-for-gestational age (SGA, birth weight < 10. percentile), duration of hospitalisation, duration of NICU stay, duration of oxygen supplementation, appearance of neonatal morbidities (i.e. NEC, early/late onset sepsis, infant respiratory distress syndrome, bronchopulmonary dysplasia, retinopathy of prematurity, intra−/periventricular haemorrhage, periventricular leucomalacia, spontaneous intestinal perforation), application of caffeine and surfactant, as well as inotropes and cardiovascular supportive therapy.
- *Data on specific medication*: dosage, active pharmaceutical ingredient and time of application of antibiotics, antifungal agents and probiotics, as well as parenteral or enteral nutrition.

Data will be collected in order to prove correlations to specific intestinal microbiota patterns. SKK, CME, GG, and BR will have access to the final trial dataset.

### Analysis of intestinal microbiota

Microbial DNA will be extracted from stool samples using a combination of mechanical and enzyme-based lysis [39]. From extracted DNA, 16S rRNA genes will be amplified with suitable primers (specific for *bacteria*, *fungi*, and *archaea*) and prepared for MiSeq Sequencing [40], which will be performed at the Core Facility for Molecular Biology, Medical University Graz for bacteria and fungi, and at the Interactive Microbiome Research Group, Medical University Graz for *archea*. Analysis will be performed using public analysis-pipelines, like Mothur (mothur.org) and Qiime (qiime.org) [41], representing open-source bioinformatics pipelines for performing microbiome analysis from raw DNA sequencing data. After a qualitative filtering process, the remaining sequences will be assigned to phylogenetic taxa and grouped into operational taxonomic units (OTU) using specific databases. Furthermore, chimeras and sequences containing sequencing artefacts will be removed.

In the frame of the Microbiome Initiative Graz (MIGrobeZ, including groups around C. Högenauer, G. Gorkiewicz and C. Moissl-Eichinger), the necessary expertise in order to analyse and interpret the data is available. All tools, pipelines, and expert knowledge of the Microbiome Initiative will be made available.

### Statistical analysis

Statistical analysis will focus on confidence interval estimation. Since the study has not been powered any hypothesis testing will be considered entirely exploratory. Full analysis will be performed in close co-operation with the core facility for bio-informatics and biostatistics, ZMF, Medical University Graz.

### Time schedule

The project is scheduled to last 24 months. At the Division of Neonatology, Department of Paediatrics, Medical University of Graz, we treat about 100 infants of < 1500 g birth weight per year. At the Division of Neonatology, Department of Paediatrics, General Hospital of Klagenfurt, between 40 and 60 infants < 1500 g birth weight are treated per year, and at the Division of Neonatology, Department of Paediatrics, General Hospital of Leoben around 30 infants < 1500 g birth weight are treated annually. Therefore, the active recruitment and treatment period regarding the study is planned to take place throughout the first 18 months. Stool microbiota analysis will take place throughout the following 2 months, and the final 4 months will be used for the statistical and biostatistical analyses of data, and for the presentation of data (abstracts, meetings, scientific papers).

## Discussion

Different surroundings at the three participating study centres, including contacts to caretakers and parents, as well as feeding or medication all might influence intestinal microbiota composition and abundance. In the planned sequel study, this should be kept in mind and a more standardised process (in particular involving random allocation) ought to be established. However, the results obtained from the presented pilot study will display the burden of bias and help to establish a more strict protocol for the future.

## Additional files

Additional file 1: SPIRIT 2013 checklist. (DOC 120 kb)

Additional file 2: SPIRIT flow-chart. (DOC 53 kb)

## Abbreviations

DNA: Desoxyrubonucleic acid
DRKS: Deutsches Register Klinischer Studien (German registry for clinical studies)
NEC: Necrotizing enterocolitis
NICU: Neonatal intensive care unit
OUT: Operational taxonomic units
RNA: Ribonucleic acid
SFD: Small for date
SOP: Standardised operating procedure
Spp.: Species
VLBW: Very low birth weight
ZMF: Zentrum für Medizinische Forschung (centre for medical sciences)
