# Supplementary material for: Changes of intestinal microbiota composition and diversity in very low birth weight infants related to strategies of NEC prophylaxis: protocol for an observational multicentre pilot study
Source: Pilot Feasibility Stud. 2017 Nov 7;3:52. doi: 10.1186/s40814-017-0195-y (PMC5678711; doi:10.1186/s40814-017-0195-y)
Supplement: Supplementary file 2 — SPIRIT flow-chart. (DOC 53 kb) [file 40814_2017_195_MOESM2_ESM.doc]

Figure…: SPIRIT Flow-Chart

|  |  |  | | **STUDY PERIOD** | | | | | | | | |
| --- | --- | --- | --- | --- | --- | --- | --- | --- | --- | --- | --- | --- |
|  | **Enrolment** | | **Allocation** | |  |  | **Post-allocation** | | | | | **Close-out** |
| **TIMEPOINT**** | ***-t_1_*** | | **0** | | ***t_1_*** | ***t_2_*** | ***t_3_*** | ***t_4_*** | ***t_5_*** | ***t_6_*** | ***t_7_*** | ***t_x_*** |
| **ENROLMENT:** |  | |  | |  |  |  |  |  |  |  |  |
| **Eligibility screen** | X | |  | |  |  |  |  |  |  |  |  |
| **Informed consent** | X | |  | |  |  |  |  |  |  |  |  |
| ***Preparation of documentations*** | X | |  | |  |  |  |  |  |  |  |  |
| **Allocation** |  | | X | |  |  |  |  |  |  |  |  |
| **INTERVENTIONS:** |  | |  | | X | X | X | X | X | X | X |  |
| ***Sample collection study group A*** |  | |  | | X | X | X | X | X | X | X |  |
| ***Sample collection study group B*** |  | |  | | X | X | X | X | X | X | X |  |
| ***Sample collection study group C*** |  | |  | | X | X | X | X | X | X | X |  |
| **ASSESSMENTS:** |  | |  | |  |  |  |  |  |  |  |  |
| ***Bacteria analysis*** |  | |  | |  |  |  |  |  |  |  | X |
| ***Archea analysis*** |  | |  | |  |  |  |  |  |  |  | X |
| ***Biostatistical and statistical analysis*** |  | |  | |  |  |  |  |  |  |  | X |

Leg.: t_1_ = day of life 1, t_2_ = day of life 2, t_3_ = day of life 3, t_4_ = day of life 4, t_5_ = day of life 5, t_6_ = day of life 6, t_7_ = day of life 7.
